# Changes to the financial responsibility for juvenile court ordered psychiatric evaluations reduce inpatient services utilization: an interrupted time series study

**DOI:** 10.1186/1472-6963-12-136

**Published:** 2012-05-30

**Authors:** Richard A Epstein, Jeff Feix, Patrick G Arbogast, Stephen H Beckjord, William V Bobo

**Affiliations:** 1 Department of Psychiatry, School of Medicine, Vanderbilt University, 1500 21st Avenue South, Village at Vanderbilt Suite 2200, Nashville, TN, 37212, USA; 2 Tennessee Department of Mental Health, 10th Floor Andrew Johnson Tower, 710 James Robertson Parkway, Nashville, TN, 37243, USA; 3 Departments of Biostatistics and Preventive Medicine, School of Medicine, Vanderbilt University, 1500 21st Avenue South, Village at Vanderbilt, Nashville, TN, 37212, USA; 4 993 Brodhead Road, Suite 202, Moon Township, PA, 15108, USA

**Keywords:** Interrupted time series, Juvenile court ordered psychiatric evaluation, Youth

## Abstract

**Background:**

The purpose of the current study was to evaluate the impact of a July 2008 Tennessee Court of Appeals opinion that shifted financial responsibility for juvenile court ordered psychiatric evaluations from the State to the County.

**Methods:**

We used de-identified administrative data from the Tennessee Department of Mental Health and mid-year population estimates from the U.S. Census Bureau from July 1, 2006 to June 30, 2010, and an interrupted time series design with segmented regression analysis to quantify the impact of the implementation of the Court opinion.

**Results:**

In the study period, there were 2,176 referrals for juvenile court ordered psychiatric evaluations in Tennessee; of these, 74.1% were inpatient evaluations. The Court opinion was associated with a decrease of 9.4 (95% C.I. = 7.9–10.8) inpatient and increase of 1.2 (95% C.I. = 0.4–2.1) outpatient evaluations per 100,000 Tennessee youth aged 12 to 19 years per month.

**Conclusions:**

The Court opinion that shifted financial responsibility for juvenile court ordered psychiatric evaluations from the State to the County was associated with a sudden and significant decrease in inpatient psychiatric evaluations, and more modest increase in outpatient evaluations.

## Background

Many youth in juvenile detention have psychiatric disorders [1]. It is therefore critical that juvenile court judges have the ability to order psychiatric evaluations to determine youth competence to stand trial and to develop appropriate service plans for youth with psychiatric disorders. Although juvenile court ordered psychiatric evaluations have traditionally been conducted on an inpatient basis, it is widely recognized that many states have moved or are moving towards conducting more of these evaluations in outpatient settings [2]. Outpatient evaluations are conducted in the community, either in a detention center or at the clinician’s office and do not require transport or admission to a psychiatric facility. Inpatient evaluations are conducted after a juvenile has been admitted to a psychiatric facility, which is typically a very restrictive locked setting and may be even higher security for juveniles charged with serious offenses. The high cost of inpatient psychiatric evaluations, the long period of time required to complete inpatient evaluations, restrictions to individual liberty that may occur when inpatient evaluations are ordered unnecessarily, and the concern that admission to a psychiatric hospital may limit an individual’s access to counsel are some of the commonly cited reasons for this general trend [3-5].

In a July 1, 2008 opinion that was implemented on September 1, 2008, the Tennessee Court of Appeals opined that financial responsibility for the direct costs of juvenile court ordered inpatient psychiatric evaluations belonged to the County rather than the State regardless whether the juvenile was charged with a misdemeanor or felony offense [6]. By historical precedent, the State had previously borne the costs of evaluations for felony-level offenses and, because juvenile courts in Tennessee are funded through County budgets, there was little incentive for juvenile court judges to consider the cost of the evaluations on felony-level offenses as long as the State was paying. Because services to provide both inpatient and outpatient psychiatric evaluations were in place before and after the Tennessee Court of Appeals issued its opinion, the opinion’s issuance provides an unusual opportunity to evaluate the effect of shifting financial responsibility for the direct costs of juvenile court ordered psychiatric evaluations for youth with a felony level offense in Tennessee from the State to the County on the use of inpatient versus outpatient services.

It is important to quantify the effect of this healthcare policy on services utilization so that policymakers can evaluate the impact of their decision on this population of youth at increased risk for receipt of suboptimal care. Thus, we hypothesized that the July 1, 2008 Tennessee Court of Appeals opinion would be associated with a decrease in the use of inpatient juvenile court ordered psychiatric evaluations and an increase in the use of outpatient juvenile court ordered psychiatric evaluations in Tennessee.

## Methods

To assess the effect of the Court of Appeals opinion on the prevalence of juvenile court ordered psychiatric evaluations, we used an interrupted time-series design [7]. Interrupted time series designs are the standard for the evaluation of policy changes which are unfeasible to study using randomized designs [8].

We used de-identified data from the Tennessee Department of Mental Health and mid-year population estimates from the U.S. Census Bureau for this study. Administrative data from the Tennessee Department of Mental Health was used to estimate the number of juvenile court ordered inpatient and outpatient psychiatric evaluations in Tennessee. Publicly available data from the U.S. Census Bureau was used to estimate the number of 12 to 19 year olds in Tennessee.

Study month was the unit of analysis. The study time period included the 48 months from July 1, 2006, through June 30, 2010, divided into 3 time periods. The period before the Court of Appeals opinion included the 24 months from July 1, 2006, to June 30, 2008. The transition period was defined as the 2 months between when the Court of Appeals opinion was issued (e.g., July 1, 2008) and when it went into effect (e.g., August 31, 2008). The period after the Court of Appeals opinion included the 22 months from September 1, 2008, to June 30, 2010.

For each study month, we calculated the prevalence of juvenile court ordered inpatient and outpatient psychiatric evaluations per 100,000 youth (aged 12 to 19 years) in the Tennessee population. Prevalence rates were calculated using data from the Tennessee Department of Mental Health on the number of juvenile court ordered inpatient and outpatient psychiatric evaluations in Tennessee during the study time period, and U.S. Census data to estimate the number of youth who were 12 to 19 years old in Tennessee [9,10]. Given the small number of events in each study month and the need to protect against the possibility of deductive identification of individual youth, demographic information was not available for inclusion in the current study.

Separate segmented linear regression models were fit for inpatient and outpatient evaluations. The regression models included a term for the effect of the implementation of the Court of Appeals opinion and adjusted for linear trends over time prior to the Court opinion and post-opinion, as well as the 2-month transition period. Correlograms were used to assess serial autocorrelation and, since there was no detectable autocorrelation, the data were modeled assuming independence. The study was reviewed and approved by the Tennessee Department of Mental Health and the Vanderbilt University Institutional Review Board.

## Results

In the 4-year study period, there were 2,176 referrals for juvenile court ordered psychiatric evaluations among youth with a felony level offense in Tennessee; of these, 74.1% were inpatient evaluations. Before the Court opinion went into effect there was a mean prevalence of 9.5 (95% CI = 8.9–10.2) inpatient and 0.9 (95% CI = 0.6–1.1) outpatient juvenile court ordered psychiatric evaluations per 100,000 Tennessee youth aged 12 to 19 years. After the Court’s opinion went into effect, the mean prevalence of inpatient evaluations was 0.1 (95% CI = 0.0–0.2) and of outpatient evaluations was 2.7 (95% CI = 2.3–3.1) per 100,000 Tennessee youth aged 12 to 19 years.

**Figure 1:**
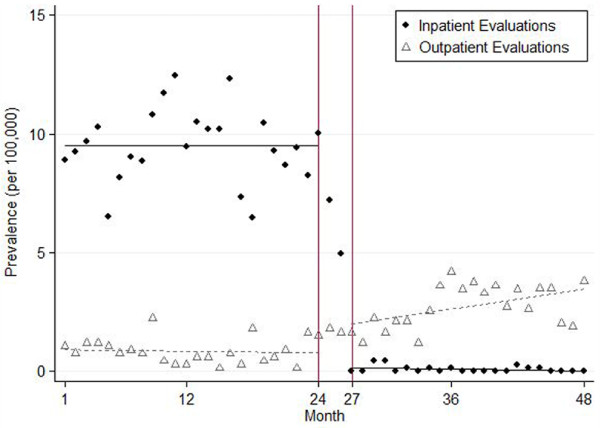
Prevalence of juvenile court ordered psychiatric evaluations per 100,000 youth aged 12 to 19 years: Tennessee, July 1, 2005 – June 30, 2010.

Segmented regression analysis shows that the Court of Appeals opinion was associated with a decrease of 9.4 inpatient evaluations (95% C.I. = 7.9–10.8) and increase of 1.2 outpatient evaluations (95% C.I. = 0.4–2.1) per 100,000 Tennessee youth aged 12 to 19 years per month (Figure 1). There were no pre- or post-opinion secular trends for inpatient evaluations. There was a slight post-opinion increasing trend of 0.8 (95% C.I. = 0.1–1.4) outpatient evaluations per 100,000 Tennessee youth aged 12 to 19 years per month.

## Discussion

Results indicate that the Tennessee Court of Appeals opinion that shifted financial responsibility for the direct costs of juvenile court ordered psychiatric evaluations for youth with a felony level offense in Tennessee from the State to the County was associated with a sudden and substantial decrease in the prevalence of juvenile court ordered inpatient psychiatric evaluations, and a modest corresponding increase in the prevalence of outpatient juvenile court ordered psychiatric evaluations. In the context of existing literature indicating that cost is an important determinant of court ordered psychiatric evaluations [2-5], the reduction in the prevalence of inpatient psychiatric evaluations after the Court of Appeals opinion is unsurprising, though the magnitude of this reduction in a span of two months is striking. It is also noteworthy that the increase in the prevalence of outpatient evaluations after the Court of Appeals opinion did not simply result in the replacement of inpatient with outpatient psychiatric evaluations. Rather, the overall prevalence of juvenile court ordered psychiatric evaluations decreased.

The magnitude of the shift in service utilization patterns between the time period prior to the Court of Appeals opinion and the time period after the Court of Appeals opinion is concerning. The implication is that inpatient services were being over-utilized before the Court of Appeals opinion, are being under-utilized after the Court of Appeals opinion, or both. The current study cannot determine which of these implications is most likely. Multiple factors are probably at play, such as the possibility that some inpatient evaluations may have been ordered to provide a type of detention to ensure the safety of the community rather than out of a genuine need for psychiatric services, and the relative unfamiliarity of the juvenile courts with the outpatient evaluation services which, although available for many years, were little used prior to the Court of Appeals decision.

The current study has several limitations. First, although interrupted time series designs are the strongest research design available to evaluate policy effects when randomization is unavailable, they cannot definitively distinguish policy effects from secular trends [8]. Second, study data did not include information on demographic variables such as age, gender, race/ethnicity or county. Given a large denominator population that is relatively stable over time, such as that used to calculate prevalence estimates in the current study, interrupted time series designs are not subject to confounding by demographic variables such as age, gender and race/ethnicity [8], but because these data are unavailable we are unable to report estimates adjusted for the effect of these variables or to test for effect modification. Anecdotal evidence suggests that there may be important and potentially confounding differences across individual counties such as whether or not the county has access to a juvenile detention center. Third, study data did not include information on the type and severity of youth mental health need. There have been many calls for juvenile courts to implement standardized mental health screening and such data, if it had been available, could have been used to answer questions about potential unmet need [11,12].

Nevertheless, the current study suggests that the September 1, 2008 implementation of the July 1, 2008 Tennessee Court of Appeals opinion that shifted financial responsibility for the direct costs of juvenile court ordered psychiatric evaluations for youth with a felony level offense in Tennessee from the State to the County was associated with a sudden and significant decrease in the prevalence of inpatient psychiatric evaluations and a modest increase in the prevalence of outpatient psychiatric evaluations. Continued monitoring of the use of these important services is warranted to more fully understand the impact of the Court of Appeals opinion on the use of juvenile court ordered psychiatric evaluations in Tennessee.

## Conclusions

The Tennessee Court of Appeals opinion that shifted financial responsibility for the direct costs of juvenile court ordered psychiatric evaluations for youth with a felony level offense in Tennessee from the State to the County was associated with a significant decrease in inpatient juvenile court ordered psychiatric evaluations and a more modest increase in outpatient evaluations. Future research examining the impact of financial changes or other policy decisions on service utilization patterns must include measures of youth need for services in order to estimate the extent to which their implementation may be associated with unmet need for services.

## Competing interests

RAE, JF, SHB and PGA declare that they have no competing interests. In the past five years, WVB has received grants/research support from Cephalon, Inc., and has served on speaker panels for Janssen Pharmacuetica and Pfizer, Inc.

## Authors’ contributions

All authors have fulfilled conditions required for authorship. RAE was involved in the conception, design, analysis, and drafting of the manuscript. JF was involved in the conception and drafting of the manuscript. PGA was involved in manuscript design and analysis. SHB was involved in the manuscript conception and design. WVB was involved in the design, analysis, and drafting of the manuscript. All authors revised the manuscript for intellectual content and approved the manuscript’s final, submitted version.

## Pre-publication history

The pre-publication history for this paper can be accessed here:

http://www.biomedcentral.com/1472-6963/12/136/prepub
